# Better Quality of Life of Peritoneal Dialysis compared to Hemodialysis over a Two-year Period after Dialysis Initiation

**DOI:** 10.1038/s41598-019-46744-1

**Published:** 2019-07-16

**Authors:** Hee-Yeon Jung, Yena Jeon, Yeongwoo Park, Yon Su Kim, Shin-Wook Kang, Chul Woo Yang, Nam-Ho Kim, Ji-Young Choi, Jang-Hee Cho, Sun-Hee Park, Chan-Duck Kim, Yong-Lim Kim

**Affiliations:** 1 Department of Internal Medicine, School of Medicine, Kyungpook National University, Kyungpook National University Hospital, Daegu, South Korea; 2 Clinical Research Center for End Stage Renal Disease, Daegu, South Korea; 3 Department of Statistics, Kyungpook National University, Daegu, South Korea; 4 Department of Internal Medicine, Seoul National University College of Medicine, Seoul, South Korea; 5 Department of Internal Medicine, Yonsei University College of Medicine, Seoul, South Korea; 6 Department of Internal Medicine, The Catholic University of Korea College of Medicine, Seoul, South Korea; 7 Department of Internal Medicine, Chonnam National University Medical School, Gwangju, South Korea; 8 Bk21 Plus KNU Biomedical Convergence Program, Department of Biomedical Science, Kyungpook National University, Daegu, South Korea

**Keywords:** Renal replacement therapy, Quality of life

## Abstract

This study aimed to compare health-related quality of life (HRQOL) over time in patients initiating hemodialysis (HD) or peritoneal dialysis (PD). A total of 989 incident patients starting HD or PD were included from a prospective nationwide cohort study. HRQOL was assessed 3, 12, and 24 months after the start of dialysis. The scores of questionnaires were adjusted for clinical and socioeconomic parameters. The adjusted three months scores of patients on PD showed better HRQOL in eight end-stage renal disease (ESRD), three physical component summary and one mental component summary domains compared with patients on HD. Both patients on HD and PD experienced significant decreases in different HRQOL domains over two years and the degree of changes in HRQOL over time was not different between dialysis modality. However, the scores of three (effects of kidney disease, burden of kidney disease, and dialysis staff encouragement, all *P* < 0.05) and two (sexual function and dialysis staff encouragement, all *P* < 0.05) ESRD domains were still higher in patients on PD compared with patients on HD at one and two years after initiation of dialysis, respectively. PD shows better HRQOL during the initial period after dialysis even after adjusting for clinical and socioeconomic characteristics, and the effect lasts up to two years. It was similar in terms of changes in HRQOL over time between HD and PD.

## Introduction

Patients with end-stage renal disease (ESRD) who are about to start long-term dialysis therapy are faced with the question of dialysis modality choice. Hemodialysis (HD) and peritoneal dialysis (PD) differ technically and in the type of effort required from patients. Barring medical contraindications and restrictions in finances or government health system for dialysis modality, patient preference should have a major role in modality selection. Although physicians mainly focus on the survival of a given modality rather than health-related quality of life (HRQOL), it is an inevitable fact of actual clinic practice that patients with ESRD care more about how they will live instead of how long^1,2^. Furthermore, a recent study have demonstrated that patients on PD and their caregivers give priority to patient-reported outcomes, such as fatigue, flexibility with time, ability to travel, sleep, and ability to work^3^. Therefore, it is crucial to give detailed information and advice regarding HRQOL regarding each dialysis modality to patients who are facing dialysis modality choice.

Previous cross-sectional studies have attempted to evaluate the differences in HRQOL among prevalent patients on HD and PD^4–8^. A few prospective studies have compared the HRQOL over time between incident patients starting HD and PD^9–13^. Most of former studies included 200–400 incident patients on HD and PD with follow-up duration of 12–18 months after dialysis initiation^10–12^. Wu *et al*. published a study regarding changes in HRQOL of 928 patients on dialysis during a one-year duration in 2004^9^. Updated information is needed on changes in HRQOL according to dialysis modality with extended follow-up period after dialysis initiation. Further, owing to the conflicting results of previous studies, there is no clear and simple answer to the question of which dialysis modality can be expected to provide better quality of life. Therefore, this study aimed to compare HRQOL over time in almost 1,000 patients initiating HD or PD from a prospective nationwide cohort study during the two-year period after dialysis initiation. The primary object of this study was to compare HRQOL over time between dialysis modality (HD versus PD) and within dialysis modality. The secondary object of this study was to determine the associated factors related to persistently impaired HRQOL in patients on dialysis.

## Results

### Characteristics of patients

**Figure 1:**
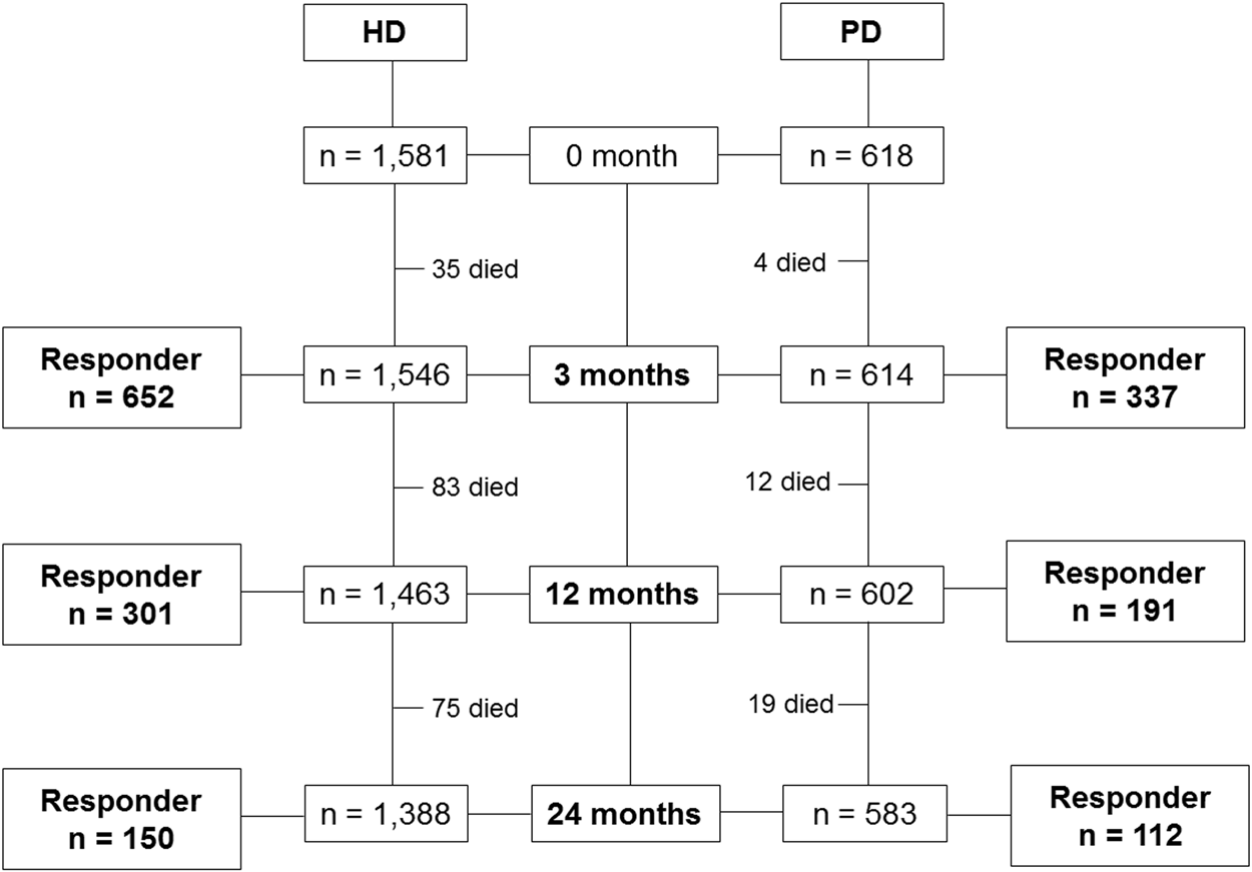
Patient flow. Among the 2,160 survivors (1,546 on HD and 614 on PD) at 3 months after dialysis initiation, 989 patients (45.8%, 652 (42.2%) on HD and 337 (54.9%) on PD) completed the questionnaire. At 12 months, among the 2,065 survivors (1,463 on HD and 602 on PD), 492 patients (23.8%, 301 (20.6%) on HD and 191 31.7%) on PD) completed the questionnaire. At 24 months, among the 1,971 survivors (1,388 on HD and 583 on PD), 262 patients (13.3%, 150 (10.8%) on HD and 112 (19.2%) on PD) completed the questionnaire. Abbreviations: HD, hemodialysis; PD, peritoneal dialysis

Figure 1 shows patient flow. Among the 2,160 survivors (1,546 on HD and 614 on PD) at 3 months after dialysis initiation, 989 patients (45.8%, 652 (42.2%) on HD and 337 (54.9%) on PD) completed the questionnaire. At 12 months, among the 2,065 survivors (1,463 on HD and 602 on PD), 492 patients (23.8%, 301 (20.6%) on HD and 191 31.7%) on PD) completed the questionnaire. At 24 months, among the 1,971 survivors (1,388 on HD and 583 on PD), 262 patients (13.3%, 150 (10.8%) on HD and 112 (19.2%) on PD) completed the questionnaire.

Table 1 shows the sociodemographic information and clinical and biochemical data of included patients at each time point. At three months, patients receiving PD were significantly younger, more educated, employed, and married but had lower modified Charlson comorbidity index (CCI) compared with patients receiving HD. Patients on PD had significantly lower serum albumin levels and higher lipid profiles than patients on HD. No difference was observed in the residual renal function (RRF) between the two groups. Among 625 patients on HD, 268 (41.1%) and 384 (58.9%) patients were enrolled from secondary and tertiary hospitals, respectively. Among 337 patients on PD, 122 (36.2%) and 215 (63.8%) patients were enrolled from secondary and tertiary hospitals, respectively.

**Table 1:** Sociodemographic, clinical, and biochemical characteristics at 3, 12, and 24 months after starting therapy according to dialysis modality.

	3 months			12 months			24 months		
	HD (n = 652)	PD (n = 337)	*P* value	HD (n = 301)	PD (n = 191)	*P* value	HD (n = 150)	PD (n = 112)	*P* value
Age (years)	56.6 ± 13.5	51.6 ± 12.8	< 0.001	56.7 ± 13.8	52.6 ± 12.4	< 0.001	57.1 ± 13.5	52.9 ± 11.8	0.009
Male sex, n (%)	409 (62.7)	201 (59.6)	0.34	187 (62.1)	106 (55.5)	0.14	95 (63.3)	65 (58.0)	0.38
Body mass index (kg/m^2^)	22.7 ± 3.2	22.9 ± 3.2	0.28	22.8 ± 3.1	23.4 ± 3.6	0.06	22.6 ± 3.1	23.9 ± 3.7	0.002
Primary renal disease, n (%)									
Diabetes	407 (62.4)	165 (49.0)	< 0.001	188 (62.5)	94 (49.2)	0.004	97 (64.7)	55 (49.1)	0.01
Non-diabetes	245 (37.6)	172 (51.0)		113 (37.5)	97 (50.8)		53 (35.3)	57 (50.9)	
Modified CCI	5.28 ± 2.24	4.64 ± 2.32	< 0.001	5.5 ± 2.1	4.7 ± 2.3	< 0.001	5.6 ± 2.2	4.8 ± 2.2	0.003
Educational level, n (%)									
<High school	260 (39.9)	110 (32.6)	0.01	103 (34.2)	68 (35.6)	0.13	52 (34.7)	38 (33.9)	0.27
High school graduate	231 (35.4)	116 (34.4)		124 (41.2)	62 (32.5)		59 (39.3)	34 (30.4)	
College graduate	155 (23.8)	110 (32.6)		71 (23.6)	60 (31.4)		38 (25.3)	39 (34.8)	
Unknown	6 (0.9)	1 (0.3)		3 (1.0)	1 (0.5)		1 (0.7)	1 (0.9)	
Employment status, n (%)									
Unemployed	477 (73.2)	207 (61.4)	< 0.001	236 (78.4)	123 (64.4)	< 0.001	112 (74.7)	71 (63.4)	0.05
Employed	175 (26.9)	130 (38.6)		65 (21.6)	68 (35.6)		38 (25.3)	41 (36.6)	
Marital status, n (%)									
Married	451 (69.2)	260 (77.2)	0.01	204 (67.8)	146 (76.4)	0.17	100 (66.7)	83 (74.1)	0.16
Widowed	65 (10.0)	17 (5.0)		30 (10.0)	10 (5.2)		15 (10.0)	3 (2.7)	
Divorced	44 (6.8)	15 (4.4)		18 (6.0)	11 (5.8)		11 (7.3)	10 (8.9)	
Not married	88 (13.5)	45 (13.4)		47 (15.6)	24 (12.6)		23 (15.3)	16 (14.3)	
Unknown	4 (0.6)	0 (0)		2 (0.7)	0 (0)		1 (0.7)	0 (0)	
Laboratory data									
Hemoglobin (g/dL)	10.7 ± 1.4	10.7 ± 1.9	0.85	10.5 ± 1.1	10.7 ± 1.7	0.20	10.5 ± 1.2	10.4 ± 1.6	0.69
Albumin (g/dL)	3.7 ± 0.5	3.4 ± 0.6	< 0.001	3.9 ± 0.4	3.5 ± 0.5	< 0.001	4.0 ± 0.4	3.5 ± 0.6	< 0.001
Calcium (mg/dL)	8.4 ± 0.8	8.3 ± 0.8	0.08	8.6 ± 0.8	8.5 ± 0.9	0.29	8.7 ± 0.9	8.7 ± 0.9	0.76
Phosphate (mg/dL)	5.2 ± 4.8	4.7 ± 3.8	0.13	4.8 ± 1.4	4.7 ± 1.3	0.55	4.9 ± 1.4	5.0 ± 1.3	0.53
LDL (mg/dL)	83.5 ± 29.0	101.5 ± 32.7	< 0.001	78.2 ± 25.4	95.4 ± 30.8	< 0.001	79.0 ± 26.7	96.8 ± 34.0	< 0.001
Triglycerides (mg/dL)	125.5 ± 72.2	144.4 ± 90.5	< 0.001	119.4 ± 66.8	146.2 ± 102.7	0.002	120.7 ± 64.2	139.5 ± 93.1	0.07
Total cholesterol (mg/dL)	152.4 ± 38.0	182.0 ± 42.6	< 0.001	146.8 ± 37.6	170.3 ± 40.9	< 0.001	146.0 ± 35.8	167.3 ± 38.0	< 0.001
Transferrin saturation (%)	31.1 ± 16.5	35.0 ± 38.0	0.07	33.4 ± 34.4	33.94 ± 12.8	0.82	31.8 ± 15.2	36.0 ± 38.1	0.27
RRF (ml/min/1.73 m^2^)	10.7 ± 1.2	11.1 ± 29.2	0.82	5.7 ± 0.7	5.8 ± 5.3	0.88	4.2 ± 0.4	4.2 ± 3.7	0.80
Hospital, n (%)									
Secondary	268 (41.4)	122 (36.2)							
Tertiary	384 (58.9)	215 (63.8)							
	*P* = 0.94	*P* = 0.08							

Values are shown as mean ± standard deviation.

Abbreviations: CCI, Charlson comorbidity index; HD, hemodialysis; PD, peritoneal dialysis; LDL, low-density lipoprotein; RRF, residual renal function.

The baseline characteristics between the responders and non-responders at 3, 12, and 24 months according to dialysis modality are presented in the Supplementary Table 1. No significant differences in age, sex, body mass index (BMI), primary renal disease, and employment status were observed between the responders and non-responders. There were significant differences in modified CCI, marital status, and some laboratory parameters between the responders and non-responders depending on the time points and dialysis modality.

### Comparison of adjusted HRQOL and BDI scores between dialysis modality

Table 2 shows the differences in HRQOL and Beck Depression Inventory (BDI) scores adjusted for age, sex, modified CCI, educational level, employment status, marital status, and hemoglobin, albumin, and total cholesterol levels between patients on HD and PD. At three months after dialysis initiation, both adjusted the kidney disease composite summary (KDCS) score and a physical composite summary (PCS) scores were significantly higher in PD patients than in HD patients (HD vs. PD: 67.4 ± 12.5 vs. 71.1 ± 12.1, *P* < 0.001 and 55.4 ± 21.3 vs. 59.1 ± 21.7, *P* = 0.04, respectively). Compared with HD patients, PD patients had significantly higher adjusted mean scores at three months in 11 domains of symptoms (HD vs. PD: 79.7 vs. 82.0, *P* = 0.04), effects of kidney disease (HD vs. PD: 68.5 vs. 73.6, *P* < 0.001), burden of kidney disease (HD vs. PD: 31.5 vs. 38.0, *P* < 0.001), work status (HD vs. PD: 25.3 vs. 37.1, *P* = 0.001), cognitive function (HD vs. PD: 83.6 vs. 85.9, *P* = 0.01), quality of social interaction (HD vs. PD: 65.5 vs. 68.5, *P* = 0.02), social support (HD vs. PD: 59.9 vs. 63.6, *P* = 0.03), dialysis staff encouragement (HD vs. PD: 85.4 vs. 88.4, *P* = 0.001), role-physical (HD vs. PD: 40.4 vs. 46.5, *P = *0.03), pain (HD vs. PD: 69.3 vs. 73.7, *P* < 0.001), and general health (HD vs. PD: 35.9 vs. 39.3, *P* = 0.03). At 12 months, the adjusted mean scores in the three domains of effects of kidney disease (HD vs. PD: 70.4 vs. 75.4, *P* = 0.005), burden of kidney disease (HD vs. PD: 32.1 vs. 38.4, *P* = 0.01), and dialysis staff encouragement (HD vs. PD: 84.1 vs. 88.0, *P* = 0.006) were still significantly higher in PD patients than in HD patients. At 24 months, PD patients had significantly higher mean scores in the two domains of sexual function (HD vs. PD: 60.9 vs. 75.0, *P* = 0.04) and dialysis staff encouragement (HD vs. PD: 84.4 vs. 88.6, *P* = 0.01).

**Table 2:** Quality of life and BDI scores at 3, 12, and 24 months after starting therapy according to dialysis modality.

	3 months		12 months		24 months	
	HD (n = 652)	PD (n = 337)	HD (n = 301)	PD (n = 191)	HD (n = 150)	PD (n = 112)
KDCS	67.4 (12.5)	71.1 (12.1)^‡^	68.2 (13.0)	71.7 (11.6)^†^	67.2 (12.4)	69.0 (12.7)
Symptom	79.7 (15.6)	82.0 (14.8)^*^	81.4 (15.0)	82.6 (12.9)	79.6 (15.6)	80.8 (14.8)
Effects of kidney disease	68.5 (18.2)	73.6 (17.5)^‡^	70.4 (18.6)	75.4 (16.2)^†^	69.8 (17.8)	72.8 (18.1)
Burden of kidney disease	31.5 (21.6)	38.0 (24.2)^‡^	32.1 (23.9)	38.4 (23.5)^*^	30.5 (22.8)	31.9 (22.4)
Work status	25.3 (32.8)	37.1 (37.4)^†^	25.1 (33.6)	36.9 (38.5)	23.7 (31.6)	32.1 (37.9)
Cognitive function	83.6 (19.0)	85.9 (16.3)^*^	84.6 (18.4)	87.1 (17.0)	82.7 (19.5)	84.2 (16.8)
Quality of social interaction	65.5 (20.4)	68.5 (19.4)^*^	66.4 (18.1)	69.2 (17.8)	68.0 (18.9)	67.3 (16.0)
Sexual function	70.3 (32.4)	74.2 (30.5)	68.5 (30.2)	74.8 (27.8)	60.9 (30.9)	75.0 (31.7)^*^
Sleep	68.1 (19.7)	69.0 (18.3)	67.2 (20.1)	69.4 (19.2)	66.6 (17.5)	67.3 (18.8)
Social support	59.9 (24.6)	63.6 (23.2)^*^	60.0 (22.7)	62.0 (22.7)	60.1 (21.8)	58.3 (21.8)
Dialysis staff encouragement	85.4 (18.5)	88.4 (14.8)^†^	84.1 (19.3)	88.0 (14.5)^†^	84.4 (16.3)	88.6 (14.2)^*^
Patient satisfaction	68.4 (23.2)	68.8 (22.1)	67.8 (22.4)	71.1 (19.3)	65.2 (23.6)	66.8 (20.7)
PCS	55.4 (21.3)	59.1 (21.7)^*^	60.5 (22.6)	60.6 (22.8)	59.3 (21.0)	59.7 (22.3)
Physical functioning	68.3 (26.0)	71.6 (26.0)	71.7 (26.5)	72.7 (26.6)	71.1 (23.6)	73.5 (25.5)
Role-physical	40.4 (41.2)	46.5 (42.7)^*^	55.2 (42.8)	51. 6 (44.5)	50.7 (43.1)	52.5 (42.0)
Pain	69.3 (24.7)	73.7 (23.2)^‡^	72.4 (23.4)	72.1 (23.7)	72.4 (23.8)	73.3 (23.9)
General health	35.9 (18.3)	38.3 (19.7)^*^	37.7 (20.2)	38.8 (19.6)	37.3 (20.5)	32.7 (18.4)
MCS	52.1 (19.8)	54.0 (20.2)	55.5 (19.5)	56.3 (19.3)	53.7 (19.7)	52.9 (21.1)
Emotional wellbeing	52.6 (17.1)	56.0 (17.4)^‡^	54.7 (16.8)	55.5 (15.6)	52.3 (17.0)	52.4 (17.3)
Role-emotional	55.4 (44.9)	55.4 (45.7)	62.6 (44.4)	63.7 (44.0)	62.4 (43.9)	60.4 (45.3)
Social function	64.0 (25.6)	65.4 (26.1)	67.5 (25.3)	70.1 (25.0)	64.4 (25.9)	66.2 (25.3)
Energy/fatigue	43.2 (18.8)	44.9 (19.1)	45.3 (17.5)	44.7 (17.2)	43.6 (17.1)	41.3 (18.0)
BDI	15.7 (10.7)	14.7 (9.8)	15.6 (10.2)	14.5 (9.5)	16.7 (9.4)	16.0 (9.4)

Values are shown as mean (standard deviation).

Abbreviations: BDI, Beck Depression Inventory; HD, hemodialysis; KDCS, kidney disease composite summary; MCS, mental composite summary; PD, peritoneal dialysis; PCS, physical composite summary.

*P* value; adjusted for age, sex, modified Charlson comorbidity index, educational level, employment status, marital status, Hb, Albumin, Total cholesterol.

^*^ Indicates *P* < 0.05 vs. HD, ^†^ indicates *P* < 0.01 vs. HD, ^‡^ Indicates *P* < 0.001 vs. HD.

No differences were observed in the BDI scores at 3, 12, and 24 months between the two groups.

### Changes in HRQOL scores over time within and between dialysis modality

**Figure 2:**
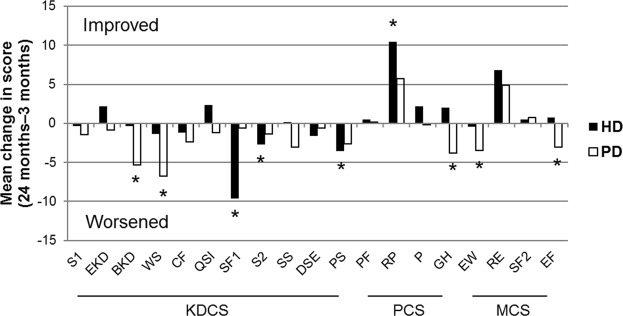
Mean changes in health-related quality of life scores from 3 to 24 months after starting therapy according to dialysis modality. Patients undergoing HD showed significantly worsened HRQOL, shown in mean changes in score, in three ESRD domains, namely, sexual function (−9.6, *P* = 0.005), sleep (−2.7, *P* = 0.04), and patient satisfaction (−3.5, *P* = 0.04), but improved HRQOL in one PCS domain, namely, role-physical (10.4, *P* = 0.002). Patients receiving PD experienced significantly worsened HRQOL, shown in mean changes in score, in two ESRD domains, namely, burden of kidney disease (−5.3, *P* = 0.009) and work status (−6.8, *P* = 0.03), in one PCS domain, namely, general health (−3.8, *P* = 0.02), and two MCS domains, namely, emotional wellbeing (−3.4, *P* = 0.02) and energy/fatigue (−3.1, *P* = 0.04). Abbreviations: BKD, burden of kidney disease; CF, cognitive function; DSE, dialysis staff encouragement; EF, energy/fatigue; EKD, effects of kidney disease; EW, emotional wellbeing; GH, general health; HD, hemodialysis; KDCS, kidney disease composite summary; MCS, mental composite summary; P, pain; PCS, physical composite summary; PD, peritoneal dialysis; PF, physical functioning; PS, patient satisfaction; QSI, quality of social interaction; RE, role-emotional; RP, role-physical; S1, symptom; S2, sleep; SF1, sexual function; SF2, social function; SS, social support; WS, work status. ^*^ Indicates *P* < 0.05 in mean changes in health-related quality of life scores from 3 to 24 months.

Figure 2 shows the mean changes in HRQOL scores in 262 patients (150 on HD and 112 on PD) from 3 to 24 months after dialysis initiation within each dialysis modality. Patients on HD experienced significantly worsened HRQOL, as indicated by their mean changes in score, in the three ESRD domains of sexual function (−9.6, *P* = 0.005), sleep (−2.7, *P* = 0.04), and patient satisfaction (−3.5, *P* = 0.04) but improved HRQOL in one PCS domain, role-physical (10.4, *P* = 0.002). Patients on PD underwent significantly worsened HRQOL, as indicated by their mean changes in score, in the two ESRD domains of burden of kidney disease (−5.3, *P* = 0.009) and work status (−6.8, *P* = 0.03); in one PCS domain, general health (−3.8, *P* = 0.02); and two a mental composite summary (MCS) domains, emotional wellbeing (−3.4, *P* = 0.02) and energy/fatigue (−3.1, *P* = 0.04).

Table 3 demonstrates the changes in HRQOL scores from 3 to 24 months after dialysis initiation between dialysis modality. No significant differences in changes in HRQOL over time were observed between dialysis modality.

**Table 3:** Changes in quality of life BDI scores over time.

	3 months		24 months		Repeated-measures ANOVA *P* (24-3 months)		
	HD	PD	HD	PD	Between-Groups Effect	Within-Groups (Time) Effect	Interaction (Group × Time) Effect
Number of patients	150	112	150	112			
KDCS	66.7 (11.4)	71.4 (10.2)	67.2 (12.4)	69.0 (12.7)	0.01	0.21	0.14
Symptom	79.1 (14.2)	84.3 (11.3)	79.6 (15.6)	80.8 (14.8)	0.04	0.16	0.11
Effects of kidney disease	66.3 (19.0)	72.9 (16.9)	69.8 (17.8)	72.8 (18.1)	0.01	0.20	0.24
Burden of kidney disease	29.8 (22.1)	35.0 (24.2)	30.5 (22.8)	31.9 (22.4)	0.21	0.40	0.16
Work status	21.1 (31.4)	38.8 (37.8)	23.7 (31.6)	32.1 (37.9)	< 0.001	0.34	0.07
Cognitive function	83.8 (18.1)	86.1 (18.1)	82.7 (19.5)	84.2 (16.8)	0.34	0.28	0.97
Quality of social interaction	66.2 (20.7)	67.9 (18.3)	68.0 (18.9)	67.3 (16.0)	0.91	0.64	0.43
Sexual function	69.9 (32.0)	87.5 (20.6)	60.9 (30.9)	75.0 (31.7)	0.01	0.24	0.61
Sleep	68.7 (19.3)	70.2 (16.6)	66.6 (17.5)	67.3 (18.8)	0.58	0.04	0.85
Social support	61.4 (23.1)	58.3 (21.8)	60.1 (21.8)	58.3 (21.8)	0.23	0.63	0.63
Dialysis staff encouragement	88.5 (15.3)	90.0 (13.6)	84.4 (16.3)	88.6 (14.2)	0.03	0.02	0.20
Patient satisfaction	69.3 (23.1)	68.6 (21.5)	65.2 (23.6)	66.8 (20.7)	0.70	0.06	0.44
PCS	55.5 (20.3)	60.8 (20.4)	59.3 (21.0)	59.7 (22.3)	0.30	0.33	0.08
Physical functioning	68.9 (23.2)	75.7 (22.6)	71.1 (23.6)	73.5 (25.5)	0.14	0.92	0.15
Role-physical	41.5 (42.7)	48.7 (42.7)	50.7 (43.1)	52.5 (42.0)	0.34	0.04	0.42
Pain	68.7 (22.9)	74.8 (21.4)	72.4 (23.8)	73.3 (23.9)	0.12	0.54	0.19
General health	34.7 (18.9)	35.0 (18.9)	37.3 (20.5)	32.7 (18.4)	0.23	0.91	0.05
MCS	50.2 (20.8)	52.9 (20.5)	53.7 (19.7)	52.9 (21.1)	0.77	0.26	0.24
Emotional wellbeing	50.6 (18.4)	54.3 (17.8)	52.3 (17.0)	52.4 (17.3)	0.41	0.96	0.14
Role-emotional	53.5 (44.5)	56.3 (46.7)	62.4 (43.9)	60.4 (45.3)	0.93	0.06	0.51
Social function	60.8 (26.1)	65.5 (25.2)	64.4 (25.9)	66.2 (25.3)	0.27	0.23	0.54
Energy/fatigue	41.9 (19.5)	42.5 (19.0)	43.6 (17.1)	41.3 (18.0)	0.52	0.86	0.29
BDI	16.4 (9.7)	15.3 (9.3)	16.7 (9.4)	16.0 (9.4)	0.41	0.37	0.69

Values are shown as mean (standard deviation).

Abbreviations: BDI, Beck Depression Inventory; HD, hemodialysis; KDCS, kidney disease composite summary; MCS, mental composite summary; PD, peritoneal dialysis; PCS, physical composite summary.

Supplementary Tables 2 and 3 show the results of HRQOL scores after adjusting the impact of hospital (secondary, tertiary) using a multilevel analysis. There were no major differences in the overall results of HRQOL.

### Analysis of factors associated with persistently low and high HRQOL scores

Table 4 gives the sociodemographic, clinical, and biochemical characteristics of patients with persistently high or low KDCS, PCS, and MCS scores at all time points. The group with persistently low KDCS had significantly higher proportion of HD patients and higher modified CCI compared with the group with persistently high KDCS. Patients with persistently low PCS were significantly older, less educated, less employed, and had higher CCI compared with patients with persistently high PCS. The group with persistently low MCS included a significantly higher proportion of men than the group with persistently high MCS. Patients with persistently low HRQOL showed significantly lower BDI scores at all time points compared with patients with persistently high HRQOL.

**Table 4:** Comparison of sociodemographic, clinical, and biochemical characteristics between patients with persistently high and low quality of life scores.

	Persistently high KDCS	Persistently low KDCS	*P* value	Persistently high PCS	Persistently low PCS	*P* value	Persistently high MCS	Persistently low MCS	*P* value
Sores at 3 months	78.5 ± 6.3	58.5 ± 8.4	< 0.001	76.0 ± 9.4	35.7 ± 13.1	< 0.001	71.2 ± 8.6	28.6 ± 12.7	< 0.001
Scores at 12 months	80.0 ± 6.1	58.0 ± 8.8	< 0.001	78.6 ± 8.2	35.6 ± 13.6	< 0.001	73.2 ± 9.0	35.3 ± 13.5	< 0.001
Scores at 24 months	78.6 ± 5.5	55.6 ± 9.9	< 0.001	78.0 ± 7.8	32.1 ± 15.0	< 0.001	70.9 ± 9.0	29.8 ± 12.7	< 0.001
Dialysis modality, n (%)									
HD	35 (50.0)	38 (70.4)	0.02	41 (54.0)	30 (55.6)	0.86	36 (58.1)	24 (53.3)	0.63
PD	35 (50.0)	16 (29.6)		35 (46.1)	24 (44.4)		26 (41.9)	21 (46.7)	
BDI scores at 3 months	8.6 ± 6.1	24.0 ± 9.5	< 0.001	10.4 ± 7.2	22.4 ± 9.4	< 0.001	9.5 ± 6.4	22.8 ± 9.7	< 0.001
BDI scores at 12 months	9.1 ± 5.5	22.9 ± 8.7	< 0.001	9.8 ± 6.3	21.8 ± 9.5	< 0.001	9.3 ± 6.4	22.8 ± 9.8	< 0.001
BDI scores at 24 months	9.7 ± 6.0	24.3 ± 8.7	< 0.001	10.8 ± 6.6	23.8 ± 9.2	< 0.001	9.3 ± 6.4	24.1 ± 7.9	< 0.001
Age (years)	50.3 ± 12.4	53.5 ± 14.2	0.19	48.9 ± 11.3	58.6 ± 12.9	< 0.001	51.2 ± 12.5	53.1 ± 15.7	0.48
Male sex, n (%)	41 (58.6)	38 (70.4)	0.18	43 (56.6)	32 (59.3)	0.76	28 (45.2)	32 (71.1)	0.008
Body mass index (kg/m^2^)	23.5 ± 3.3	22.5 ± 3.3	0.10	23.0 ± 3.2	22.0 ± 3.3	0.08	23.2 ± 3.1	21.7 ± 3.5	0.03
Primary renal disease, n (%)									
Diabetes	40 (57.1)	34 (63.0)	0.51	40 (52.6)	34 (63.0)	0.24	38 (61.3)	25 (55.6)	0.67
Non-diabetes	30 (42.9)	20 (37.0)		36 (47.4)	20 (37.0)		24 (38.7)	20 (44.4)	
Modified CCI	4.6 ± 2.1	5.5 ± 2.2	0.03	4.2 ± 1.8	6.2 ± 2.2	< 0.001	4.7 ± 2.1	5.4 ± 2.4	0.14
Educational level, n (%)									
< High school	13 (18.6)	17 (31.5)	0.14	16 (21.1)	26 (48.2)	0.003	18 (29.0)	11 (24.4)	0.67
High school graduate	31 (44.3)	23 (42.6)		31 (40.8)	15 (27.8)		24 (38.7)	20 (44.4)	
College graduate	26 (37.1)	13 (24.1)		29 (38.2)	12 (22.2)		20 (32.3)	13 (28.9)	
Unknown	0 (0)	1 (1.9)		0 (0)	1 (1.9)		0 (0)	1 (2.2)	
Employment status, n (%)									
Unemployed	40 (57.1)	40 (74.1)	0.05	46 (60.5)	45 (83.3)	0.005	43 (69.4)	31 (68.9)	0.96
Employed	30 (42.9)	14 (25.9)		30 (39.5)	9 (16.7)		19 (30.7)	14 (31.1)	
Marital status, n (%)									
Married	49 (70.0)	32 (59.3)	0.39	56 (73.7)	39 (72.2)	0.16	46 (74.2)	30 (66.7)	0.48
Widowed	4 (5.7)	5 (9.3)		1 (1.3)	4 (7.4)		3 (4.8)	1 (2.2)	
Divorced	6 (8.6)	3 (5.6)		4 (5.3)	5 (9.3)		5 (8.1)	3 (6.7)	
Unknown	11 (15.7)	14 (25.9)		15 (19.7)	6 (11.1)		8 (12.9)	11 (24.4)	
Laboratory data									
Hemoglobin (g/dL)	10.8 ± 1.3	10.6 ± 1.4	0.51	10.8 ± 1.2	10.6 ± 1.5	0.48	10.8 ± 1.2	10.7 ± 1.1	0.53
Albumin (g/dL)	3.6 ± 0.5	3.7 ± 0.5	0.51	3.7 ± 0.5	3.5 ± 0.5	0.14	3.6 ± 0.5	3.6 ± 0.5	0.80
Calcium (mg/dL)	8.3 ± 0.8	8.1 ± 0.7	0.23	8.2 ± 0.8	8.3 ± 0.9	0.89	8.2 ± 0.8	8.2 ± 0.9	0.71
Phosphate (mg/dL)	4.9 ± 1.4	4.8 ± 1.4	0.57	5.0 ± 1.6	4.6 ± 1.2	0.15	4.7 ± 1.6	4.7 ± 1.4	0.90
LDL (mg/dL)	88.0 ± 26.1	93.8 ± 43.7	0.38	93.9 ± 30.4	100.0 ± 41.2	0.35	94.1 ± 29.8	96.5 ± 43.0	0.75
Triglycerides (mg/dL)	131.2 ± 103.1	143.5 ± 69.9	0.43	136.3 ± 107.3	136.9 ± 66.5	0.97	129.4 ± 64.5	139.3 ± 67.8	0.44
Total cholesterol (mg/dL)	163.3 ± 37.3	164.7 ± 56.0	0.87	173.3 ± 41.9	168.7 ± 49.0	0.57	168.5 ± 38.3	169.1 ± 52.0	0.95
Transferrin saturation (%)	31.5 ± 10.6	30.9 ± 15.8	0.83	32.5 ± 12.1	29.9 ± 10.7	0.21	32.1 ± 11.9	32.6 ± 13.4	0.83
RRF at 1 year (ml/min/1.73 m^2^)	5.7 ± 2.8	5.3 ± 1.3	0.28	5.7 ± 2.6	5.1 ± 1.9	0.10	6.2 ± 4.4	5.5 ± 5.1	0.45
RRF at 2 year (ml/min/1.73 m^2^)	4.43 ± 2.3	4.0 ± 0.8	0.13	4.3 ± 2.3	3.9 ± 1.0	0.24	4.2 ± 2.3	4.4 ± 4.6	0.82

Values are shown as mean ± standard deviation.

Abbreviations: CCI, Charlson comorbidity index; HD, hemodialysis; KDCS, kidney disease composite summary; LDL, low-density lipoprotein; MCS, mental composite summary; PCS, physical composite summary; PD, peritoneal dialysis; RRF, residual renal function.

From the multivariate analysis (Table 5), a high BDI score at three months was an independent risk factor for persistently low KDCS (odds ratio [OR] = 1.34, 95% confidence interval [CI]: 1.21–1.49, *P* < 0.001), PCS (OR = 1.21, 95% CI: 1.12–1.31, *P* < 0.001), and MCS (OR = 1.26, 95% CI: 1.15–1.37, *P* < 0.001). High modified CCI (OR = 1.44, 95% CI: 1.08–1.93, *P* = 0.01) and unemployment (OR = 5.27, 95% CI: 1.48–18.70, *P* = 0.01) were significantly associated with persistently low PCS.

**Table 5:** Multivariate logistic regression analysis results for factors associated with persistently low KDCS, PCS, and MCS.

	Persistently low KDCS		Persistently low PCS		Persistently low MCS	
	OR (95% CI)	*P* value	OR (95% CI)	*P* value	OR (95% CI)	*P* value
Dialysis modality						
HD	3.05 (0.88–0.61)	0.08	0.63 (0.21–1.88)	0.41	0.93 (0.31–2.76)	0.90
PD	reference		reference		reference	
BDI score at 3 months	1.34 (1.21–1.49)	< 0.001	1.21 (1.12–1.31)	< 0.001	1.26 (1.15–1.37)	< 0.001
Age (years)	1.02 (0.96–1.07)	0.57	1.04 (0.99–1.1)	0.11	1.00 (0.95–1.06)	0.91
Modified CCI	1.01 (0.75–1.36)	0.94	1.44 (1.08–1.93)	0.01	1.03 (0.73–1.45)	0.87
Employment status						
Unemployed	0.97 (0.29–3.22)	0.97	5.27 (1.48–18.70)	0.01	0.71 (0.22–2.26)	0.56
Employed	reference		reference		reference	

Abbreviations: BDI, Beck Depression Inventory; CCI, Charlson comorbidity index; CI, confidence interval; HD, hemodialysis; KDCS, kidney disease composite summary; MCS, mental composite summary; OR, odds ratio; PCS, physical composite summary; PD, peritoneal dialysis.

## Discussion

This prospective nationwide cohort study compared HRQOL at 3, 12, and 24 months after dialysis initiation using KDQOL-SF^TM^ 1.3 between patients starting HD and PD and investigated detailed changes in HRQOL over time. Although the HRQOL advantage of PD tended to decrease with time, the effect was found to last up to two years after dialysis, even after adjusting for clinical and socioeconomic characteristics. In terms of two-year changes in HRQOL over time within dialysis modality, both patients on HD and PD experienced a worsening of HRQOL in different domains. During a two-year period after dialysis initiation, while patients on HD underwent significantly worsened sexual function, sleep, and patient satisfaction, patients on PD experienced significantly increased burden of kidney disease and work status and worsened general health, emotional wellbeing, and mental energy. The degree of changes in HRQOL over time was not different between dialysis modality.

The results of previous studies, mainly cross-sectional ones, were not conclusive regarding which dialysis modality could ensure a better HRQOL. Several studies have reported advantages of PD in some domains of HRQOL^4,6,12,14^, advantages of HD^5,11^, and no differences between two dialysis modality^7–9^. This disagreement on the results of previous studies might be due to the differences in characteristics of included patients, such as dialysis vintage, comorbidities, and socioeconomic conditions, and the type of instrument used in assessing HRQOL. Our main findings show that patients initiating PD had better HRQOL scores in several domains compared with patients starting HD in the initial period after dialysis, even after adjusting for background imbalances in characteristics between HD and PD. Patients on PD were bothered less by the burden of ESRD, symptoms, and pain, and were able to continue their jobs more compared with those on HD. These results can be explained by the fundamental differences in dialysis method between HD and PD. Unlike patients on PD, patients undergoing HD have to go to dialysis centers two to three times a week for four hours per session. This requirement might negatively affect both personal lives and occupational achievement. Further, patients receiving PD maintained social interaction and social support more actively, had more satisfaction with dialysis staff encouragement, and ultimately felt better general physical health and emotional wellbeing compared with those undergoing HD.

In our study, at the end of one year, patients on PD were still feeling less burdened by the disease itself. Higher satisfaction for dialysis staff encouragement was sustained in patients on PD for two years. Previous cross-sectional studies have also shown similar results with respect to advantage of PD regarding dialysis staff encouragement^4,8,15^ and treatment satisfaction^16^. This result might represent positive and important information for patients who are reluctant to undergo PD because they feel pressured to perform dialysis on their own. The benefit of dialysis staff support could be highlighted to encourage the use and availability of PD as a dialysis modality.

The notable finding in our study was that patients on HD and PD experienced a worsening of different domains in HRQOL over time. The results on the changes in HRQOL could give more valuable information than the results regarding the comparison of HRQOL at each time points between dialysis modality since we cannot completely exclude the possibility that patients with better HRQOL may choose PD as a dialysis modality. The results of longitudinal follow-up revealed that patients on HD had more problems with sexual function and sleep, as well as experienced decreased patient satisfaction over time since beginning dialysis. Only patients on HD showed significant improvement in role-physical over time. This outcome might be because patients on HD had considerably impaired baseline role-physical score (mean 40.4) and thus had more room for improvement. Meanwhile, the burden of kidney disease was on the increase, whereas emotional wellbeing and energy were on the decrease in patients on PD; the benefit of PD in terms of maintaining a job decreased over time. As such, physicians should ask different questions regarding HRQOL over time according to dialysis modality to identify and solve problems that dialysis patients are facing and to increase HRQOL eventually. Further, considering the detrimental effect of testosterone deficiency, which results in sexual dysfunction^17^, impaired sleep quality^18^, and low mental health^19^ on mortality of dialysis patients, it is also important to have proper consultation with specialists according to symptoms to improve survival. Our results regarding changes in specific aspects of HRQOL over time could provide valuable information regarding trade-offs, which may facilitate decision-making on dialysis modality to individual patients with specific preferences. A previous study has shown that comprehensive information with shared decision-making (SDM) between the patient and the doctor is needed for patients with ESRD when choosing dialysis modality^20^. Although there are no evidence-based guidelines in SDM for patients with chronic kidney disease, successful SDM has been shown to improve the quality of medical decision and treatment success, as well as reduce costs^20,21^. Hence, information on HRQOL should be fully included and reflected in SDM for dialysis choice.

In this study, depressive symptoms at the initial period after dialysis initiation had continuous detrimental effects on all aspects of HRQOL, including ESRD, MCS, and PCS domains, regardless of dialysis modality. Previous studies have also revealed that depressive symptoms and depression itself are significantly associated with impaired HRQOL^22^ and higher mortality^23^ in patients on dialysis by affecting compliance with dialysis and medication adherence, altering immune system function, and rendering a negative effect on nutritional status^24–26^. Despite the significant impact of depressive symptoms on clinical outcomes, depressive symptoms and depression are often under-recognized and undertreated in dialysis patients^27^. Therefore, it is important to account for depressive symptoms to prevent incident dialysis patients from experiencing persistent impaired HRQOL. Further studies will be required to clarify how regularly depressive symptoms and HRQOL should be evaluated in patients on dialysis as dialysis-related parameters, along with residual renal function and laboratory findings.

This study has a number of limitations. First, there is no information on measured pre-dialysis HRQOL, which might have yielded more useful results. We cannot completely exclude the possibility that HRQOL was already better in PD than HD patients before they began dialysis. However, we performed HRQOL analysis by adjusting for various confounding factors to reduce the impact of baseline clinical and socioeconomic parameters on HRQOL. Second, the response rate of the questionnaire declined over time. The non-response rate was higher in patients on HD compared with those on PD, which may affect the results of this study. This might be due to the high transfer rate to local dialysis clinic in patients on HD in South Korea. Third, considering international variation in HRQOL^28,29^, caution should be taken when generalizing and applying our findings to other ethnicities because we included only an East Asian population. Forth, we have no information about the primary reason for initial dialysis modality choice which can be affected by medical and social contraindications except for patients’ own modality choice and might have an impact on HRQOL of dialysis patients. Fifth, there were significant differences in some clinical and laboratory parameters between the responders and non-responders. Interestingly, comorbidity index of non-responders was higher compared with that of responders in patients on HD and comorbidity index of non-responders was lower compared with that of responders in patients on PD. Therefore, we speculate that if more patients completed the follow-up surveys, better HRQOL of PD would have been clearer. Sixth, the follow-up period was not long enough to demonstrate gradual deterioration of RRF in PD patients and show its association with HRQOL in PD patients. Nevertheless, our study has strengths in that it showed detailed changes in HRQOL over time according to dialysis modality, with a relatively large number of incident patients on dialysis and with a longer duration of follow-up compared with previous prospective longitudinal studies evaluating HRQOL over time between incident patients starting HD and PD^9–12^.

In conclusion, PD shows better HRQOL during the initial period after dialysis even after adjusting for clinical and socioeconomic characteristics, and the effect lasts up to two years. Both patients on HD and PD experienced a worsening of HRQOL over time in different domains. It was similar in terms of changes in HRQOL over time between HD and PD. It may mean that there is no dialysis modality which has definite advantage in terms of changes in HRQOL over time. It is important to give detailed information and advice regarding HRQOL to patients considering dialysis modality choice. Previous studies have shown that patients are willing to abandon 15–23 months of life expectancy to obtain greater freedom to travel and 7 months to reduce hospital visits^1,2^; meanwhile, the HRQOL of patients on dialysis has received little academic attention. It is time for nephrologists and dialysis staff to pay attention to the actual preferences and priorities of dialysis patients. Therefore, regular monitoring of HRQOL and depressive symptoms, as well as appropriate intervention to specific symptoms, could be crucial for patients who are forced to take maintenance dialysis for life support.

## Methods

### Study design and participants

This study included a total of 989 incident patients starting HD or PD who completed baseline questionnaires and stayed on the same modality, aged 19 years or older, from a prospective nationwide cohort study in Korea (Clinical Research Center for End Stage Renal Disease, clinicaltrial.gov NCT00931970). All patients provided their written informed consent before participating in the study, and the institutional review board of each center approved the study protocol. [The Catholic University of Korea, Bucheon St. Mary’s Hospital; The Catholic University of Korea, Incheon St. Mary’s Hospital; The Catholic University of Korea, Seoul St. Mary’s Hospital; The Catholic University of Korea, St. Mary’s Hospital; The Catholic University of Korea, St. Vincent’s Hospital; The Catholic University of Korea, Uijeongbu St. Mary’s Hospital; Cheju Halla General Hospital; Chonbuk National University Hospital; Chonnam National University Hospital; Chung-Ang University Medical Center; Chungbuk National University Hospital; Chungnam National University Hospital; Dong-A University Medical Center; Ehwa Womans University Medical Center; Fatima Hospital, Daegu; Gachon University Gil Medical Center; Inje University Pusan Paik Hospital; Kyungpook National University Hospital; Kwandong University College of Medicine, Myongji Hospital; National Health Insurance Corporation Ilsan Hospital; National Medical Center; Pusan National University Hospital; Samsung Medical Center, Seoul; Seoul Metropolitan Government, Seoul National University, Boramae Medical Center; Seoul National University Hospital; Seoul National University, Bundang Hospital; Yeungnam University Medical Center; Yonsei University, Severance Hospital; Yonsei University, Gangnam Severance Hospital; Ulsan University Hospital; Wonju Christian Hospital (in alphabetical order)]. All clinical investigations were conducted in accordance with the guidelines of the 2008 Declaration of Helsinki.

### Variables and data collection

Sociodemographic information, clinical and biochemical data, HRQOL, and depressive symptoms were obtained 3, 12, and 24 months after the start of dialysis. The questionnaire of KDQOL-SF^TM^ 1.3 consists of 80 items divided into 19 domains plus 1 separate item that compares current health status with health status one year ago. Each domain is rated on a scale from 0 to 100, with higher scores reflecting better HRQOL. The scores of the ESRD-targeted items are aggregated into KDCS score. The scores of the 36-Item Short Form Health Survey, a patient-reported survey of patient health, are classified into PCS score that includes items related to physical function, physical roles, pain, and general health, as well as MCS score that includes items related to emotional roles, emotional wellbeing, emotional energy, and social functioning^30^. BDI-II consists of 21 self-reported items; each item is scored from 0 to 3, with the total score ranging from 0 to 63^31^. Sociodemographic information, clinical and biochemical data include age, sex, BMI, primary kidney disease (diabetes/non-diabetes), modified CCI, educational level (<high school/high school graduate/college graduate), employment status (unemployed/employed), marital status (married/widowed/divorced/not married), laboratory data (hemoglobin, albumin, calcium, phosphate, lipid profiles, and transferrin saturation), and RRF.

Participants were divided into two groups dichotomized based on the median levels of HRQOL scores at 3, 12, 24 months. The median values of KDCS at 3, 12, 24 months were 68.6, 69.6, and 67.9, respectively. The median values of PCS at 3, 12, 24 months were 56.7, 60.5, and 59.5, respectively. The median values of MCS at 3, 12, 24 months were 52.8, 55.8, and 53.4, respectively. We classified participants with persistently higher or lower scores than median values at each time point as belonging to the “persistently high” group or “persistently low” group, respectively.

### Statistical analysis

Sociodemographic, clinical, and biochemical data were compared using Pearson’s chi-squared test or Fisher’s exact test for categorical variables and using the student’s *t*-test for continuous variables. Continuous variables are expressed as mean ± standard deviation. The scores of KDQOL-SF^TM^ 1.3 and BDI-II were adjusted for age, sex, modified CCI, educational level, employment status, marital status, and hemoglobin, albumin, and total cholesterol levels. Differences in questionnaire scores at each time point between dialysis modality were analyzed from regression analyses adjusted for variables. To determine the effects of dialysis modality (HD versus PD) and time, as well as the interaction between both, we used repeated measures ANOVA. Specifically, we used generalized estimating equation models for repeated measures to examine the association of the scores of HRQOL with dialysis modality across time. Multivariate logistic regression analysis was used to investigate the association between questionnaire scores and background variables. The multivariate model was built with factors including dialysis modality, depressive symptoms, age, comorbidity index, and employment status, which were found to be associated with HRQOL^32–35^. The mean for continuous variables and mode for categorical variables of the non-missing values of each variable were used to impute the missing values. Multilevel analysis was used to adjust the impact of hospital (secondary, tertiary) on HRQOL. Statistical analysis was performed using the SAS system for Windows, version 9.2 (SAS Institute Inc., Cary, NC) and R (R Foundation for Statistical Computing, Vienna, Austria; www.r-project.org). *P* values < 0.05 were considered statistically significant.

## Supplementary information

Supplementary Information

## Data Availability

Data supporting the findings of the current study are available from the corresponding author on reasonable request.
