# Supplementary material for: Better Quality of Life of Peritoneal Dialysis compared to Hemodialysis over a Two-year Period after Dialysis Initiation
Source: Sci Rep. 2019 Jul 16;9:10266. doi: 10.1038/s41598-019-46744-1 (PMC6635359; doi:10.1038/s41598-019-46744-1)
Supplement: Supplementary file 1 — Supplementary Information [file 41598_2019_46744_MOESM1_ESM.pdf]

Better Quality of Life of Peritoneal Dialysis compared to Hemodialysis over a Two-year Period after Dialysis Initiation

Hee-Yeon Jung,[1, 2] Yena Jeon,[2, 3] Yeongwoo Park,[2, 3] Yon Su Kim,[2, 4] Shin-Wook Kang,[2, 5] Chul Woo Yang,[2, 6] Nam-Ho Kim,[2, 7] Ji-Young Choi,[1, 2] Jang-Hee Cho,[1, 2] Sun-Hee Park,[1, 2] Chan-Duck Kim,[1, 2] and Yong-Lim Kim[1, 2, 8*]

[1]Department of Internal Medicine, School of Medicine, Kyungpook National University, Kyungpook National University Hospital, Daegu, South Korea

[2]Clinical Research Center for End Stage Renal Disease, Daegu, South Korea

[3]Department of Statistics, Kyungpook National University, Daegu, South Korea

[4]Department of Internal Medicine, Seoul National University College of Medicine, Seoul, South Korea

[5]Department of Internal Medicine, Yonsei University College of Medicine, Seoul, South Korea

[6]Department of Internal Medicine, The Catholic University of Korea College of Medicine, Seoul, South Korea

[7]Department of Internal Medicine, Chonnam National University Medical School, Gwangju, South Korea

[8]Bk21 Plus KNU Biomedical Convergence Program, Department of Biomedical Science, Kyungpook National University, Daegu, South Korea

Short title: Quality of Life: HD vs. PD

[*]**Corresponding author**:

Yong-Lim Kim, MD, PhD

Professor

Division of Nephrology, Department of Internal Medicine

School of Medicine, Kyungpook National University, Kyungpook National University Hospital, Daegu, 41944, South Korea

Tel: +82-53-200-5553

Fax: +82-53-423-7583

E-mail: ylkim@knu.ac.kr

Supplementary Table S1. The comparison of sociodemographic, clinical, and biochemical characteristics between the responders and non-responders in 3, 12, and 24 months.

	3 months						12 months						24 months					
	HD			PD			HD			PD			HD			PD		
	R	Non-R	*P* value	R	Non-R	*P* value	R	Non-R	*P* value	R	Non-R	*P* value	R	Non-R	*P* value	R	Non-R	*P* value
Number of patients	652	894		337	277		301	1162		191	411		150	1238		112	471	
Age (years)	56.6 ± 13.5	58.0 ± 12.2	0.29	51.6 ± 12.8	51.7 ± 12.8	0.97	56.0 ± 13.8	57.3 ± 12.4	0.13	51.8 ± 12.4	51.1 ± 12.8	0.55	55.3 ± 13.5	56.9 ± 12.5	0.13	51.1 ± 11.8	51.0 ± 12.8	0.95
Male sex, n (%)	409 (62.7)	550 (61.5)	0.63	201 (59.6)	167 (60.3)	0.93	187 (62.1)	722 (62.1)	1.00	105 (55.3)	253 (61.6)	0.15	95 (63.3)	762 (61.6)	0.72	65 (58.0)	286 (60.7)	0.67
Body mass index (kg/m^2)	22.7 ± 3.2	22.9 ± 5.2	0.42	22.9 ± 3.2	22.9 ± 2.9	0.76	22.5 ± 3.1	22.9 ± 4.8	0.09	22.9 ± 3.2	22.9 ± 3.1	0.91	22.4 ± 3.1	23.0 ± 4.7	0.04	23.0 ± 3.3	22.9 ± 3.0	0.80
Primary renal disease, n (%)																		
Diabetes	407 (62.4)	530 (59.3)	0.23	165 (49.0)	151 (54.5)	0.19	188 (62.5)	688 (59.2)	0.32	94 (49.5)	216 (52.5)	0.48	97 (64.7)	730 (59.0)	0.19	55 (49.1)	241 (51.2)	0.75
Non-diabetes	245 (37.6)	364 (40.7)		172 (51.0)	126 (45.5)		113 (37.5)	474 (40.8)		96 (50.5)	195 (47.5)		53 (35.3)	508 (41.0)		57 (50.9)	230 (48.8)	
Modified CCI	5.3 ± 2.2	5.5 ± 2.3	0.07	4.6 ± 2.3	4.3 ± 2.1	0.04	5.3 ± 2.1	5.3 ± 2.3	0.96	4.8 ± 2.3	4.3 ± 2.1	0.01	5.4 ± 2.2	5.2 ± 2.2	0.27	4.8 ± 2.2	4.2 ± 2.2	0.02
Educational level, n (%)																		
< High school	260 (39.9)	341 (38.1)	0.05	110 (32.6)	79 (28.5)	< 0.001	105 (34.9)	444 (38.2)	0.29	67 (35.3)	116 (28.2)	0.05	52 (34.7)	457 (36.9)	0.73	38 (33.9)	134 (28.5)	0.40
High school graduate	231 (35.4)	321 (35.9)		116 (34.4)	89 (32.1)		121 (40.2)	414 (35.6)		63 (33.2)	138 (33.6)		59 (39.3)	459 (37.1)		34 (30.4)	163 (34.6)	
College graduate	155 (23.8)	206 (23.0)		110 (32.6)	94 (33.9)		72 (23.9)	278 (23.9)		59 (31.1)	142 (34.5)		38 (25.3)	298 (24.1)		39 (34.8)	159 (33.8)	
Unknown	6 (0.9)	26 (2.9)		1 (0.3)	15 (5.4)		3 (1.0)	26 (2.2)		1 (0.5)	15 (3.6)		1 (0.7)	24 (1.9)		1 (0.89)	15 (3.2)	
Employment status, n (%)																		
Unemployed	477 (73.2)	663 (74.2)	0.68	207 (61.4)	167 (60.3)	0.80	227 (75.4)	840 (72.3)	0.31	116 (61.1)	249 (60.6)	0.93	11 3(75.3)	895 (72.3)	0.50	67 (59.8)	284 (60.3)	1.00
Employed	175 (26.8)	231 (25.8)		130 (35.6)	110 (39.7)		74 (24.6)	322 (27.7)		74 (39.0)	162 (39.4)		37 (24.7)	343 (27.7)		45 (40.2)	187 (39.7)	
Marital status, n (%)																		
Married	451 (69.2)	699 (78.2)	< 0.001	260 (77.2)	211 (76.2)	0.001	207 (68.8)	881 (75.8)	0.04	146 (76.8)	313 (76.2)	0.03	100 (66.7)	927 (74.9)	0.15	84 (75.0)	361 (76.7)	0.04
Widowed	65 (10.0)	70 (7.8)		17 (5.0)	9 (3.3)		28 (9.3)	93 (8.0)		9 (4.7)	16 (3.9)		15 (10.0)	96 (7.8)		3 (2.7)	18 (3.8)	
Divorced	44 (6.8)	24 (2.7)		15 (4.5)	5 (1.8)		16 (5.3)	49 (4.2)		11 (5.8)	9 (2.2)		11 (7.3)	54 (4.4)		9 (8.0)	11 (2.3)	
Not married	88 (13.5)	84 (9.4)		45 (13.4)	42 (15.2)		48 (16.0)	122 (10.5)		24 (12.6)	63 (15.3)		23 (15.3)	144 (11.6)		16 (14.3)	71 (15.1)	
Unknown	4 (0.6)	17 (1.9)		0 (0)	10 (3.6)		2 (0.7)	17 (1.5)		0 (0)	10 (2.4)		1 (0.67)	17 (1.4)		0 (0)	10 (2.1)	
Laboratory data																		
Hemoglobin (g/dL)	10.7 ± 1.4	10.9 ± 1.2	0.003	10.7 ± 1.9	11.0 ± 1.6	0.01	10.5 ± 1.1	10.8 ± 0.9	< 0.001	10.7 ± 1.7	10.8 ± 1.4	0.19	10.5 ± 1.2	10.8 ± 0.8	< 0.001	10.4 ± 1.5	10.4 ± 1.0	0.79
Albumin (g/dL)	3.7 ± 0.5	3.7 ± 0.4	0.26	3.4 ± 0.6	3.5 ± 0.5	< 0.001	3.9 ± 0.4	4.0 ± 0.3	0.13	3.5 ± 0.5	3.6 ± 0.5	0.62	4.0 ± 0.4	4.0 ± 0.3	0.55	3.5 ± 0.6	3.6 ± 0.4	0.79
Calcium (mg/dL)	8.4 ± 0.8	8.6 ± 0.7	0.007	8.3 ± 0.8	8.5 ± 0.8	0.02	8.6 ± 0.8	8.7 ± 0.6	< 0.001	8.5 ± 0.9	8.6 ± 0.8	0.22	8.7 ± 0.8	8.9 ± 0.5	0.04	8.7 ± 0.9	8.7 ± 0.6	0.93
Phosphate (mg/dL)	5.2 ± 4.8	4.7 ± 1.2	0.79	4.7 ± 3.8	4.6 ± 1.1	0.47	4.8 ± 1.4	4.6 ± 1.0	0.43	4.7 ± 1.3	4.7 ± 1.1	0.61	4.9 ± 1.4	4.6 ± 0.8	0.004	5.0 ± 1.3	4.9 ± 1.1	0.95
LDL (mg/dL)	83.5 ± 29.0	88.3 ± 21.8	< 0.001	101.5 ± 32.7	97.0 ± 24.5	0.23	77.0 ± 25.2	78.8 ± 16.2	0.003	96.7 ± 30.6	96.2 ± 26.5	0.74	76.8 ± 26.3	77.8 ± 13.4	0.08	97.4 ± 33.9	92.0 ± 21.3	0.91
Triglycerides (mg/dL)	125.5 ± 72.1	127.8 ± 58.5	< 0.001	144.4 ± 90.5	137.3 ± 62.3	0.96	118.9 ± 66.8	121.6 ± 46.5	< 0.001	147.0 ± 102.8	143.8 ± 66.0	0.13	120.4 ± 64.2	125.7 ± 47.3	0.34	139.8 ± 93.0	137.5 ± 58.4	0.02
Total cholesterol (mg/dL)	152.4 ± 38.0	162.2 ± 34.4	< 0.001	182 ± 42.6	177.6 ± 36.0	0.19	146.6 ± 37.5	149.4 ± 23.5	< 0.001	170.7 ± 40.9	172.6 ± 33.2	0.21	145.7 ± 35.8	147.4 ± 20.3	0.05	167.3 ± 38.0	165.2 ± 28.2	0.77
Transferrin saturation (%)	31.1 ± 16.5	36.2 ± 60.6	< 0.001	33.0 ± 38.0	33.8 ± 12.2	0.31	33.5 ± 34.4	36.4 ± 27.6	< 0.001	34.3 ± 12.8	37.5 ± 26.8	0.15	32.6 ± 15.6	46.3 ± 114.3	< 0.001	36.3 ± 38.1	37.0 ± 12.7	< 0.001
RRF (ml/min/$1.73m^2$)	10.7 ±	10.8 ±	0.06	11.1 ±	10.1 ±	< 0.001	6.2 ± 0.7	6.3 ± 0.4	0.10	5.7 ± 5.3	5.3 ± 1.7	0.007	7.0 ± 0.8	7.2 ± 0.7	< 0.001	4.5 ± 3.7	4.9 ± 1.4	< 0.001

1.2	1.4	29.2	6.2

Values are shown as mean ± standard deviation.

Abbreviations: CCI, Charlson comorbidity index; HD, hemodialysis; Non-R, non-responders; PD, peritoneal dialysis; LDL, low-density lipoprotein; R, responders; RRF, residual renal function.

Table S2. Quality of life and BDI scores at 3, 12, and 24 months after starting therapy according to dialysis modality using a multilevel analysis.

	3 months			12 months			24 months		
	HD (n = 652)	PD (n = 337)	*P* value	HD (n = 301)	PD (n = 191)	*P* value	HD (n = 150)	PD (n = 112)	*P* value
KDCS	67.4 (12.5)	71.1 (12.1)	< 0.001	68.2 (13.0)	71.7 (11.6)	0.002	67.2 (12.4)	69.0 (12.7)	0.12
Symptom	79.7 (15.6)	82.0 (14.8)	0.01	81.4 (15.0)	82.6 (12.9)	0.26	79.6 (15.6)	80.8 (14.8)	0.27
Effects of kidney disease	68.5 (18.2)	73.6 (17.5)	< 0.001	70.4 (18.6)	75.4 (16.2)	0.002	69.8 (17.8)	72.8 (18.1)	0.10
Burden of kidney disease	31.5 (21.6)	38.0 (24.2)	< 0.001	32.1 (23.9)	38.4 (23.5)	0.006	30.5 (22.8)	31.9 (22.4)	0.60
Work status	25.3 (32.8)	37.1 (37.4)	< 0.001	25.1 (33.6)	36.9 (38.5)	< 0.001	23.7 (31.6)	32.1 (37.9)	0.04
Cognitive function	83.6 (19.0)	85.9 (16.3)	0.02	84.6 (18.4)	87.1 (17.0)	0.10	82.7 (19.5)	84.2 (16.8)	0.25
Quality of social interaction	65.5 (20.4)	68.5 (19.4)	0.02	66.4 (18.1)	69.2 (17.8)	0.08	68.0 (18.9)	67.3 (16.0)	0.81
Sexual function	70.3 (32.4)	74.2 (30.5)	0.36	68.5 (30.2)	74.8 (27.8)	0.36	60.9 (30.9)	75.0 (31.7)	0.05
Sleep	68.1 (19.7)	69.0 (18.3)	0.40	67.2 (20.1)	69.4 (19.2)	0.23	66.6 (17.5)	67.3 (18.8)	0.60
Social support	59.9 (24.6)	63.6 (23.2)	0.03	60.0 (22.7)	62.0 (22.7)	0.56	60.1 (21.8)	58.3 (21.8)	0.43
Dialysis staff encouragement	85.4 (18.5)	88.4 (14.8)	0.006	84.1 (19.3)	88.0 (14.5)	0.02	84.4 (16.3)	88.6 (14.2)	0.02
Patient satisfaction	68.4 (23.2)	68.8 (22.1)	0.50	67.8 (22.4)	71.1 (19.3)	0.07	65.2 (23.6)	66.8 (20.7)	0.39
PCS	55.4 (21.3)	59.1 (21.7)	0.008	60.5 (22.6)	60.6 (22.8)	0.90	59.3 (21.0)	59.7 (22.3)	0.87
Physical functioning	68.3 (26.0)	71.6 (26.0)	0.06	71.7 (26.5)	72.7 (26.6)	0.58	71.1 (23.6)	73.5 (25.5)	0.51
Role-physical	40.4 (41.2)	46.5 (42.7)	0.02	55.2 (42.8)	51. 6 (44.5)	0.42	50.7 (43.1)	52.5 (42.0)	0.67
Pain	69.3 (24.7)	73.7 (23.2)	0.003	72.4 (23.4)	72.1 (23.7)	0.94	72.4 (23.8)	73.3 (23.9)	0.64
General health	35.9 (18.3)	38.3 (19.7)	0.05	37.7 (20.2)	38.8 (19.6)	0.57	37.3 (20.5)	32.7 (18.4)	0.06
MCS	52.1 (19.8)	54.0 (20.2)	0.10	55.5 (19.5)	56.3 (19.3)	0.60	53.7 (19.7)	52.9 (21.1)	0.80
Emotional wellbeing	52.6 (17.1)	56.0 (17.4)	0.003	54.7 (16.8)	55.5 (15.6)	0.55	52.3 (17.0)	52.4 (17.3)	1.00
Role-emotional	55.4 (44.9)	55.4 (45.7)	0.77	62.6 (44.4)	63.7 (44.0)	0.61	62.4 (43.9)	60.4 (45.3)	0.86
Social function	64.0 (25.6)	65.4 (26.1)	0.35	67.5 (25.3)	70.1 (25.0)	0.28	64.4 (25.9)	66.2 (25.3)	0.57

Energy/fatigue	43.2 (18.8)	44.9 (19.1)	0.16	45.3 (17.5)	44.7 (17.2)	0.69	43.6 (17.1)	41.3 (18.0)	0.28
BDI	15.7 (10.7)	14.7 (9.8)	0.08	15.6 (10.2)	14.5 (9.5)	0.15	16.7 (9.4)	16.0 (9.4)	0.41

Values are shown as mean (standard deviation).

Abbreviations: BDI, Beck Depression Inventory; HD, hemodialysis; KDCS, kidney disease composite summary; MCS, mental composite summary; PD, peritoneal dialysis; PCS, physical composite summary.

P value; adjusted for age, sex, modified Charlson comorbidity index, educational level, employment status, marital status, Hb, Albumin, Total cholesterol

Table S3. Changes in quality of life BDI scores over time using a multilevel analysis.

	3 months		24 months		Repeated-measures ANOVA *P* (24-3 months)		
	HD	PD	HD	PD	Between-Groups Effect	Within-Groups (Time) Effect	Interaction (Group × Time) Effect
Number of patients	150	112	150	112			
KDCS	66.7 (11.4)	71.4 (10.2)	67.2 (12.4)	69.0 (12.7)	0.004	0.70	0.10
Symptom	79.1 (14.2)	84.3 (11.3)	79.6 (15.6)	80.8 (14.8)	0.01	0.61	0.07
Effects of kidney disease	66.3 (19.0)	72.9 (16.9)	69.8 (17.8)	72.8 (18.1)	0.01	0.03	0.21
Burden of kidney disease	29.8 (22.1)	35.0 (24.2)	30.5 (22.8)	31.9 (22.4)	0.15	0.68	0.22
Work status	21.1 (31.4)	38.8 (37.8)	23.7 (31.6)	32.1 (37.9)	< 0.001	0.57	0.08
Cognitive function	83.8 (18.1)	86.1 (18.1)	82.7 (19.5)	84.2 (16.8)	0.14	0.45	0.83
Quality of social interaction	66.2 (20.7)	67.9 (18.3)	68.0 (18.9)	67.3 (16.0)	0.72	0.42	0.38
Sexual function	69.9 (32.0)	87.5 (20.6)	60.9 (30.9)	75.0 (31.7)	0.006	0.43	0.60
Sleep	68.7 (19.3)	70.2 (16.6)	66.6 (17.5)	67.3 (18.8)	0.45	0.12	0.83
Social support	61.4 (23.1)	58.3 (21.8)	60.1 (21.8)	58.3 (21.8)	0.22	0.70	0.68
Dialysis staff encouragement	88.5 (15.3)	90.0 (13.6)	84.4 (16.3)	88.6 (14.2)	0.06	0.03	0.23
Patient satisfaction	69.3 (23.1)	68.6 (21.5)	65.2 (23.6)	66.8 (20.7)	0.72	0.05	0.48
PCS	55.5 (20.3)	60.8 (20.4)	59.3 (21.0)	59.7 (22.3)	0.18	0.06	0.10
Physical functioning	68.9 (23.2)	75.7 (22.6)	71.1 (23.6)	73.5 (25.5)	0.07	0.46	0.17
Role-physical	41.5 (42.7)	48.7 (42.7)	50.7 (43.1)	52.5 (42.0)	0.26	0.02	0.44
Pain	68.7 (22.9)	74.8 (21.4)	72.4 (23.8)	73.3 (23.9)	0.08	0.26	0.21
General health	34.7 (18.9)	35.0 (18.9)	37.3 (20.5)	32.7 (18.4)	0.35	0.40	0.07
MCS	50.2 (20.8)	52.9 (20.5)	53.7 (19.7)	52.9 (21.1)	0.54	0.14	0.23
Emotional wellbeing	50.6 (18.4)	54.3 (17.8)	52.3 (17.0)	52.4 (17.3)	0.29	0.96	0.13

Role-emotional	53.5 (44.5)	56.3 (46.7)	62.4 (43.9)	60.4 (45.3)	0.71	0.04	0.56
Social function	60.8 (26.1)	65.5 (25.2)	64.4 (25.9)	66.2 (25.3)	0.20	0.17	0.47
Energy/fatigue	41.9 (19.5)	42.5 (19.0)	43.6 (17.1)	41.3 (18.0)	0.73	0.73	0.25
BDI	16.4 (9.7)	15.3 (9.3)	16.7 (9.4)	16.0 (9.4)	0.28	0.71	0.78

Values are shown as mean (standard deviation).

Abbreviations: BDI, Beck Depression Inventory; HD, hemodialysis; KDCS, kidney disease composite summary; MCS, mental composite summary; PD, peritoneal dialysis; PCS, physical composite summary.
